# Regulation of P450-derived epoxy fatty acids in cardiovascular diseases

**DOI:** 10.1016/j.rbc.2025.100064

**Published:** 2025-10-14

**Authors:** Matthew L. Edin, Joan P. Graves, Darryl C. Zeldin

**Affiliations:** Division of Intramural Research, National Institute of Environmental Health Sciences, National Institutes of Health, 111 T.W. Alexander Drive, Research Triangle Park, NC, 27709, USA

**Keywords:** Cytochrome P450, Eicosanoids, Cardiovascular, Lipid oxidation, Oxylipins

## Abstract

The cytochrome P450 (P450 or CYP) superfamily, while extensively recognized for xenobiotic metabolism, is also critically involved in the biosynthesis of lipid mediators from polyunsaturated fatty acids (PUFAs). Distinct from cyclooxygenase (COX) and lipoxygenase (LOX) pathways, P450 enzymes uniquely catalyze the metabolism of PUFAs such as arachidonic acid (AA), to epoxy fatty acids (EpFAs), such as epoxyeicosatrienoic acids (EETs) and mid-chain or ω-terminal hydroxyeicosatrienoic acids (HETEs). Eicosanoid biosynthesis is initiated by phospholipase A_2_ (PLA_2_)-mediated release of PUFAs from cellular membranes. Members of the CYP1-CYP4 subfamilies, including prominent human isoforms like CYP2C8, CYP2C9, and CYP2J2, exhibit significant epoxygenase activity, yielding regio- and stereo-specific EETs. These P450-derived oxylipins exert diverse physiological effects, influencing critical processes such as vascular tone, inflammation, angiogenesis, and ischemia-reperfusion injury. Their biological actions are often modulated by soluble epoxide hydrolase (sEH) and microsomal epoxide hydrolase (mEH), which hydrolyze EETs to less active dihydroxyeicosatrienoic acids (DHETs). Furthermore, P450s metabolize other PUFAs, including linoleic acid (LA), eicosapentaenoic acid (EPA), and docosahexaenoic acid (DHA), generating distinct EpFAs with varying biological effects. Understanding the complex interplay of P450 isoforms, their substrate preferences, and the subsequent metabolic fates of their products is crucial for elucidating their functional roles in health and disease.

## 1. Introduction

**Fig. 1.**
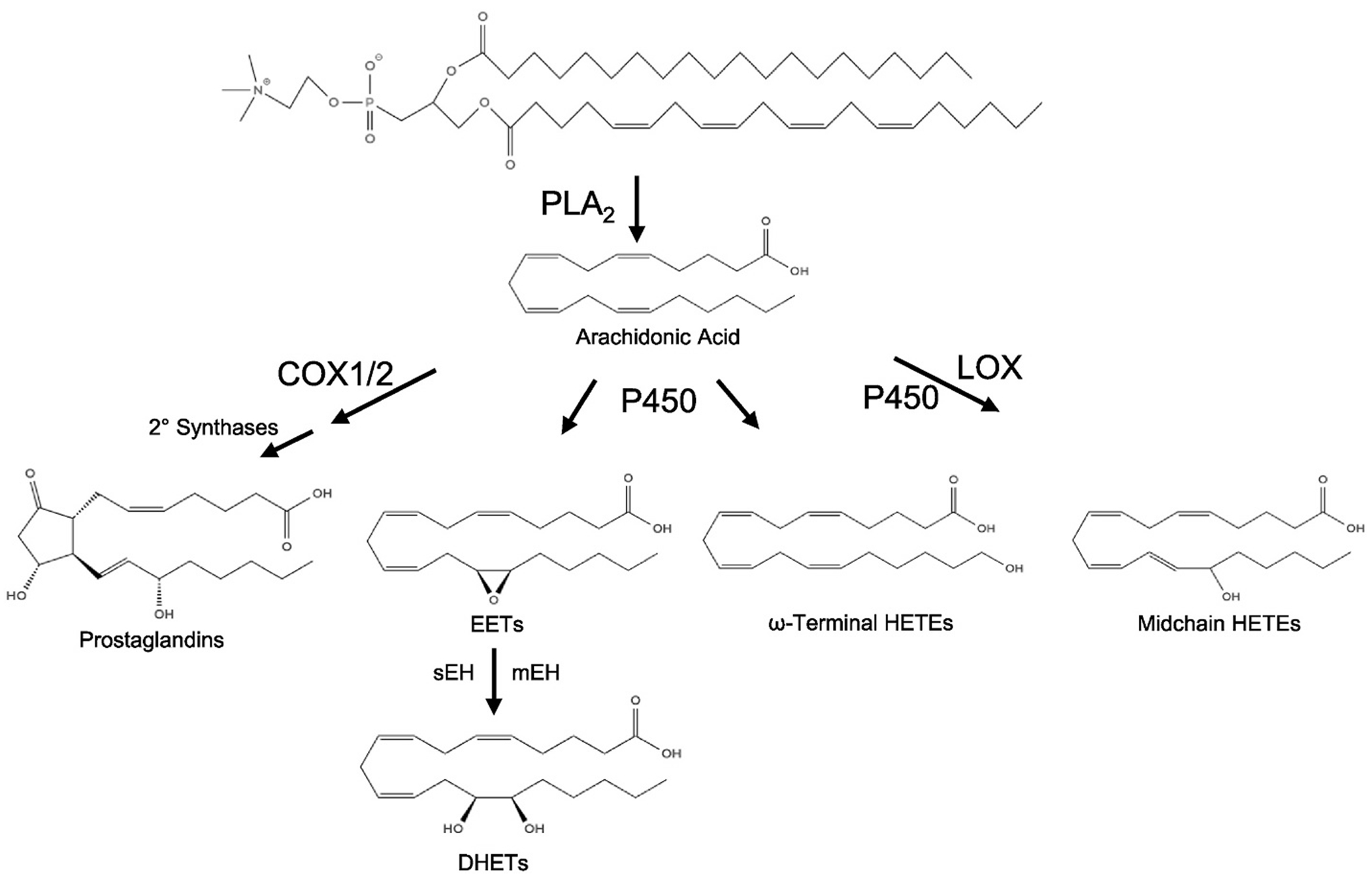
Arachidonic acid is released from membrane phospholipids by PLA_2_ and then metabolized by cyclooxygenases-1 and -2 (COX1/2) and secondary synthases to form prostaglandins (PGs), by cytochromes P450 (CYP) to form epoxyeicosatrienoic acids (EETs), ω-terminal or midchain hydroxyeicosatrienoic acids (HETEs), and by lipoxygenases (LOX) to form HETEs, leukotrienes (LTs), and lipoxins (LXs). LTs and LXs not represented.

While the cytochrome P450 (CYP) superfamily is extensively studied for its role in xenobiotic metabolism, it also plays an important role in polyunsaturated fatty acid (PUFA) metabolism. The cyclooxygenase (COX) and lipoxygenase (LOX) pathways are well known for their metabolism of the canonical PUFA arachidonic acid (AA) to biologically active prostanoids, or hydroxyeicsoatrienoic acids (HETEs), leukotrienes and lipoxins, respectively [1]. COX and LOX metabolism of AA generates eicosanoids that play important functional roles in a wide array of biological processes including inflammation, chemotaxis, cellular proliferation and intracellular signaling [1]. CYPs also metabolize AA to a variety of HETEs and are the only enzyme system that produces epoxy fatty acids (EpFAs), such as the AA-derived epoxyeicosatrienoic acids (EETs). While humans and mice have 57 and 102 P450 isoforms, respectively, only a subset of the CYP1-CYP4 subfamily enzymes oxidize fatty acids. These subfamily members metabolize AA to three types of eicosanoids (Fig. 1). Allylic oxidation forms several midchain conjugated dienols, which include 5-, 8-, 9-, 11-, 12-, and 15-hydroxyeicosatrienoic acids (HETEs). P450s can also hydroxylate AA to produce 16-, 17-, 18-, 19-, and 20-HETEs. Olefin epoxidation produces four regioisomeric *cis*-epoxyeicosatrienoic acids (14,15-, 11,12-, 8,9-, and 5,6-EETs). P450-derived eicosanoids possess many potent biological activities [2]. This review will focus on the P450s that epoxygenate PUFAs, their regulation in cardiovascular diseases and the mechanisms through which EpFAs regulate physiological processes.

## 2. Phospholipase A_2_ initiation of eicosanoid biosynthesis

The initial step in eicosanoid production by P450s is the release of PUFAs, such as AA, from cell membranes. Most PUFAs in cells are found esterified to the *sn*-2 position of cell membrane glycerophospholipids [1]. PUFA content in phospholipids can regulate membrane structure and influence the function of lipid rafts and organelles [3,4]. Phospholipid-bound PUFAs also provides a reservoir of lipid substrates during the initial step in eicosanoid biosynthesis [1,5]. Physiological stimuli such as growth factors, infection, inflammation and ischemia can activate phospholipase A_2_ (PLA_2_) enzymes that release sn-2 esterified PUFAs to make them available for oxidative metabolism [5–7].

There are approximately 20 mammalian PLA_2_ enzymes that may be involved in eicosanoid formation or release. These are subdivided into three categories based on their cellular localization and requirement for Ca^2+^. Cytosolic PLA_2_ (cPLA_2_) isoforms respond to micromolar concentrations of Ca^2+^ to translocate to the membranes to release PUFAs. Ca^2+^-independent PLA_2_ (iPLA_2_) are also expressed intracellularly and may be regulated by calmodulin, ATP, caspase cleavage, or protein aggregation. Secretory PLA_2_ (sPLA_2_) can act either intracellularly or after release into extracellular compartments where they are activated by millimolar concentrations of Ca^2+^ [8].

Disrupting PLA_2_ function inhibits eicosanoids formation. Inherited polymorphisms in *PLA2G4A* disrupt cPLA_2α_ function, attenuate eicosanoid levels in humans, and result in enteric ulcerations similar to that observed with COX inhibition or deficiency [9–11]. Similarly, cPLA_2α_ inhibition reduced prostaglandin production and inflammatory responses in mice [12]. sPLA_2_ concentrates in compartments enriched in AA-containing phospholipids, COX and/or LOX enzymes, which suggests a possible role in eicosanoid formation. Increased sPLA_2_ expression or transgenic sPLA_2_ overexpression is associated with elevated cardiovascular disease risk in humans and atherosclerosis in mice, respectively; though it is unclear if these are related to eicosanoid levels [12,13] .iPLA_2_-deficient smooth muscle cells display diminished cell proliferation and motility while iPLA_2_-deficient macrophages display altered inflammatory polarization; both effects were attributed to decreased AA release and prostaglandin production [14,15].

## 3. P450 monooxygenases

P450 enzymes are identified by their hallmark ‘pigment’ that, when carbon monoxide binds to the reduced form of the enzyme, exhibit an unusual absorption maximum at 450 nm [16]. This unusual 450 nm absorbance is a direct consequence of the unique properties of the protoporphyrin IX heme iron coordination by the highly conserved heme binding peptide (HBP). P450 HBPs conform to the consensus sequence F/G-X-G-X-R-X-C-X-G (where X can be any amino acid), with the invariant cysteine residue (C) which acts as the thiolate donor to the heme iron [16]. While many specific P450s and P450 subfamilies are highly conserved across mammalian species, gene duplication events and natural selection have resulted in a remarkable diversity of P450s [16,17]. While humans have 57 *CYP* genes, mice have 102 *Cyp* genes with many of the additional genes represented in subfamilies known to oxidize fatty acids. For example, while humans have 1 *CYP2B*, 4 *CYP2C*, 1 *CYP2J* and 4 *CYP3A* genes, mice have 5 *Cyp2b*, 15 *Cyp2c*, 7 *Cyp2j* and 8 *Cyp3a* genes [17]. While some well-conserved human *CYP* genes such as *CYP1A1, CYP1A2* or *CYP2E1* have direct homologs with similar names in mice (*Cyp1a1, Cyp1a2*, and *Cyp2e1*), other orthologous subfamilies do not have clear homologs. For example, none of the 7 murine *Cyp2j* isoforms stand out as a conclusive homolog to human *CYP2J2*; by convention, these were named sequentially by order of discovery (*Cyp2j5, Cyp2j6, Cyp2j8, Cyp2j9, Cyp2j11, Cyp2j12* and *Cyp2j13*). This expansion may not be related to enhanced diversity of eicosanoid formation, as CYP1A, CYP2B, CYP2C, CYP2J, and CYP3A subfamilies are responsible for the majority of xenobiotic and pharmaceutical metabolism [18,19].

The structure and catalytic cycle of P450s involved in the metabolism of fatty acids to oxylipins has been expertly reviewed elsewhere [16]. All the fatty acid monooxygenases discussed herein catalyze the insertion of one atom of molecular oxygen into the substrate (RH) while the other oxygen atom is reduced to water, following the general stoichiometry:

$$\text{RH}+{\text{O}}_{2}+\text{NADPH}+\text{H}+\to \text{ROH}+{\text{H}}_{2}\text{H}+\text{NADP}+$$

At difference with lipoxygenases and prostaglandin synthetases, where reactions involve initiation to substrate by carbon free radicals, P450s are unique among enzymes catalyzing AA oxidation in that product formation (epoxidation) involves oxygen reduction and activation to an “oxenoid” form and subsequent delivery to ground state C–C double bonds [20,21]. In the reaction, reduced nicotinamide adenine dinucleotide diphosphate (NADPH) is oxidized to NADP + by an enzyme called either cytochrome P450 reductase (CPR) or P450 oxidoreductase (POR). This process provides two electrons to the heme iron in P450 to complete the reaction [16]. Some P450s can receive the second electron from cytochrome *b*5 or use cytochrome *b*5 to enhance activity [22], particularly in mice with CPR disruption [23]; however, CPR is wholly sufficient for P450 function and considered the predominant P450 reductase [19]. The P450/substrate combinations that use CPR versus cytochrome *b*5 for fatty acid oxidation have not been extensively examined.

### 3.1. P450-dependent formation of EETs

Evidence for P450-dependent generation of EETs from AA was first detected in kidney and liver microsomes [24,25]. Remarkably, from the initial finding of P450-dependent DHET formation, Oliw et al. deduced the entire pathway of P450-induced AA epoxidation and subsequent enzymatic hydrolysis [25]. P450s can incorporate oxygen into any olefin of AA to generate all four regioisomers (5,6-, 8,9-, 11,12-, and 14, 15-EET) exclusively as *cis*-EETs [24–27]. Tissue microsomes and recombinant CYP enzymes show a preference for generation of 14,15- and 11,12-EET over other regioisomers [28]. 14,15-EET is often the most abundant EET; however, in many systems, 11,12-EET has the highest activity [29–32]. Hydroperoxide-dependent CYP oxidation of arachidonic acid can form both *cis*- and *trans*-EETs which have independent signaling capacity *in vivo* [33–36].

CYPs produce all four EET regioisomers as (S,R) or (R,S) stereoisomers, with ratios varying by CYP and regioisomer. For example, CYP2C8 selectively generates 11(R),12(S)- and 14(R),15(S)-EET [17,26,37]. In contrast, CYP2J2 favors 14(R),15(S)-EET but produces equal amounts of 11,12-EET stereoisomers [38]. Though many studies use racemic mixtures, EET stereoisomers have distinct biological effects that must be determined empirically. For instance, while 14(S),15(R)-EET is better at dilating bovine coronary arteries only 11(R),12(S)-EET dilates small renal arterioles at low concentrations [39]. 14(R),15(S)-EET binds more readily to the membrane binding site on U937 cells, suggesting it may be a more potent agonist for a putative EET receptor [40].

### 3.2. P450s with AA epoxygenase activity

EETs have been shown to be generated by extracts from numerous tissues, including liver, kidney, lung, skin, heart, brain, adrenal, pituitary, ovaries, and blood vessels [25,28,38,41–48]. A variety of cell types, including cardiomyocytes, astrocytes, endothelial cells, and cancer cells can produce EETs [49–52]. These findings suggest broad expression of P450s with epoxygenase activity and many expressed human CYPs and mammalian orthologs can generate EETs. Comparison of different P450s in their metabolism of AA into EETs is challenging as many factors can influence kinetic activity. Differences in preparation and handling (freeze/thawing) of microsomes or purified enzymes, variation in concentrations of substrate or NADPH supply, and measurement by HPLC versus LC/MS may significantly affect the accuracy of reported EET formation rates. Based on homology to known EET producing enzymes, up to 16 human and 43 mouse P450s may be capable of EET biosynthesis. It is likely that there is substantial redundancy in the enzymes involved in producing EETs that influence physiological responses.

In humans, CYP2C8, CYP2C9 and CYP2J2 display the highest rates of AA metabolism and produce high percentages (>50 %) of EETs (Table 1). CYP2C8 and CYP2C9 both metabolize AA at high rates; CYP2C8 generates mostly 14,15- and 11,12-EET, while CYP2C9 produces 14,15-, 11,12- and 8,9-EET [37,53–55]. CYP2C19 generates small amounts of 14,15- and 8,9-EET, but is not considered a major human EET producer [37,53,54]. CYP2C8 is abundantly expressed in heart, liver, kidney, and intestines, but is also found in blood vessels to varying degrees [56–59]. CYP2C9 is found in liver, intestines and extrahepatic tissues and is thought to be the predominant P450 with AA epoxygenase activity in human coronary arteries and aorta [56–59]. CYP2J2 metabolizes AA to produce a high percentage of fatty acid epoxides with a preference for formation of 14,15-EET [53]. CYP2J2 is thought to be the most abundant EET producer in the heart and is also expressed in intestines, liver, kidney and blood vessels [38,57,59]. CYP3A4 metabolizes AA at a lower rate and produces less than half its products as EETs; however, CYP3A isoforms are the most abundant P450s in both liver and intestines [56,60]. In addition, CYP3A4 expression enhances hepatocyte and breast cancer cell proliferation in an EET-dependent manner, which suggests that CYP3A4 may act as a biologically relevant EET producer *in vivo* [61,62].

CYP4X1 demonstrated epoxygenase activity by metabolism of anandamide to 14,15-EET ethanolamide [63] and we observe efficient metabolism of AA to EETs (Edin and Zeldin, unpublished observations). CYP4X1 is highly expressed in the brain in several species, but is also present in the lung, liver, kidney and female reproductive tissues [63–65]. Purified CYP1A1, CYP1A2, CYP2B6 and CYP2E1 generate far more hydroxyl compounds than epoxides from AA [53,55]. While these isoforms do not appear to be significant EET producers, *in vitro* metabolism may not accurately reflect the contribution of these isoforms to *in vivo* formation. For example, omeprazole, a known inducer of CYP1A1 and CYP1A2 expression [66], synergizes with sEH inhibitors to dramatically increase plasma EETs in rats without inducing expression of CYP2C or CYP2J isoforms [67].

In addition to the isoforms listed above many additional rodent P450 homologs have been shown to be capable of EET production. Of these, at least eight murine CYP2Cs (CYP2C29, CYP2C38, CYP2C39, CYP2C40, CYP2C44, CYP2C50, CYP2C54, and CYP2C55) and 5 rat CYP2Cs (CYP2C2, CYP2C10, CYP2C11, CYP2C23, and CYP2C24) produce EETs [54,68–72]. All 7 murine CYP2Js (CYP2J5, CYP2J6, CYP2J8, CYP2J9, CYP2J11, CYP2J12, CYP2J13) and both rat CYP2Js (CYP2J3 and CYP2J4) produce EETs [54,73,74]. Mouse and rat both express a CYP4X1 homolog [56,65,75]. Other rodent CYP2 family members known to produce EETs include CYP2B1, CYP2B12, and CYP2B19 [45, 54,76]. Known rabbit epoxygenases include CYP2B4, CYP2B5, CYP2C1, CYP2C2, and possibly CYP2J1 [42,77,78] (Table 1). In most instances, assigning these orthologs as homologs to human CYP members is problematic due to variation in P450 sequences, expression patterns and/or metabolic profiles.

### 3.3. P450 hydroxylase metabolism of AA

While this review focuses on the formation, regulation and impact of P450-derived EpFAs, many P450s also induce hydroxylation of AA to hydroxyeicosatetraenoic acids (HETEs) that have important physiological effects. P450 hydroxylase reactions can be divided into two subsets: (a) those that insert oxygen distal to the omega (ω) olefin of AA (ω-hydroxylases) that produce primarily 19-, and 20-HETE [79]; and (b) midchain hydroxylases that perform a lipoxygenase-like reaction (bis-allylic oxidation) that can produce 5-, 8-, 9-, 11-, 12-, and 15-HETEs [53]. ω-Hydroxylation reactions are carried out primarily by members of the CYP4A, CYP4B, and CYP4F subfamilies [80,81], though 19- and 20-HETE are often minor products of P450s that primarily produce EpFAs [73,82]. Similarly, midchain HETEs can be minor products of P450s that primarily act as epoxygenases [68,73]. While P450-derived ω-HETEs have important effects in inflammation, vasodilation, renal function, and obesity [83], the physiological consequences of P450-derived midchain HETE formation are hard to discern. Various LOX enzymes produce 5-, 8-, 12-, and 15-HETE in large quantities [84] and COXs can produce 11- and 15-HETE under certain circumstances [85,86], making the relative contribution of these P450-derived HETEs difficult to determine.

### 3.4. Biological fate of EETs

**Fig. 2.**
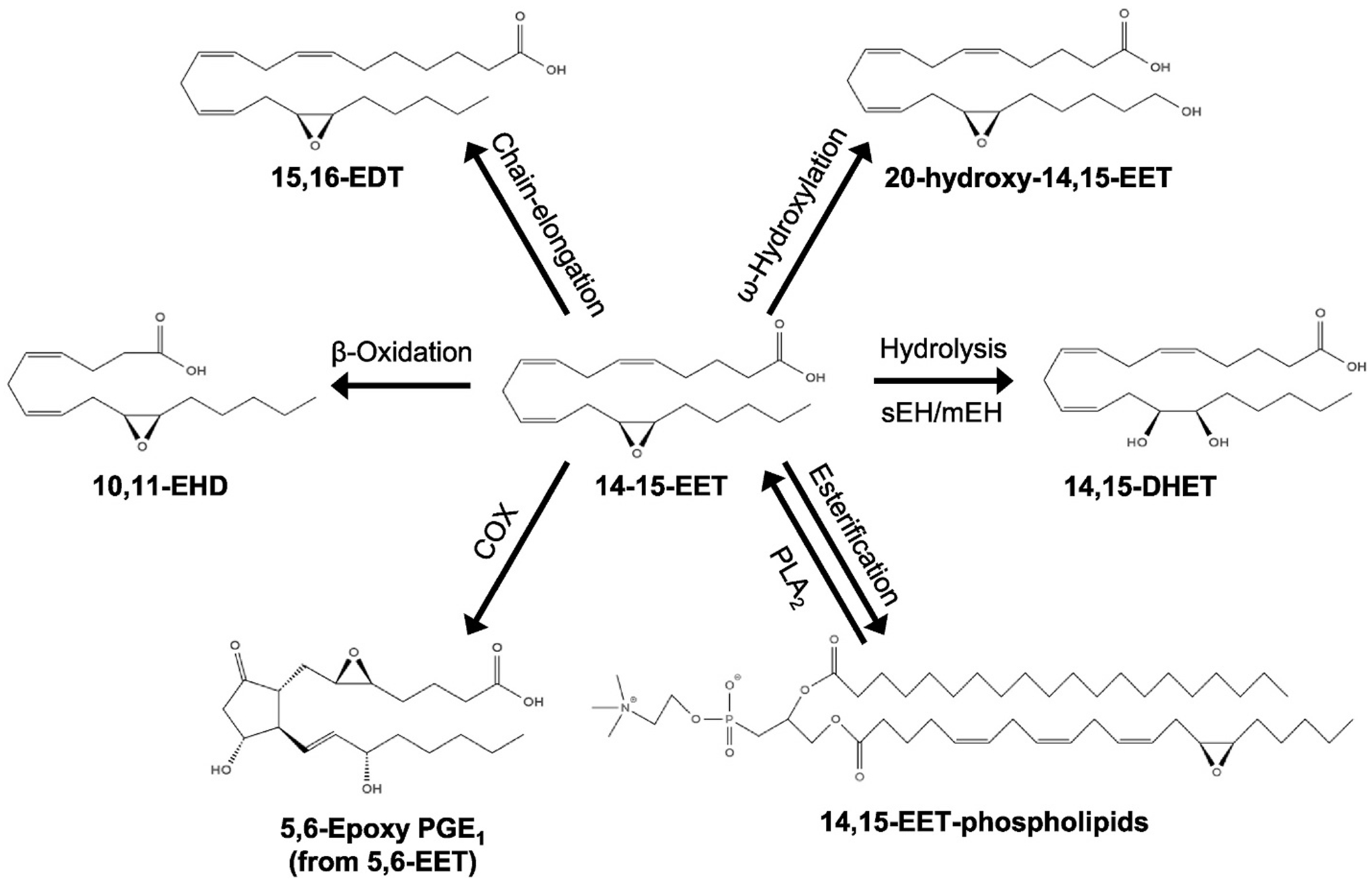
Fatty acid epoxides, such as 14,15-epoxyeicosatrienoic acid (14,15-EET), are metabolized by multiple pathways. EETs can undergo additional oxygenation by CYPs or cyclooxygenases (COXs), can be hydrolyzed to DHETs by soluble or microsomal epoxide hydrolase (sEH or mEH), or become esterified to the membrane phospholipids in an acyl-CoA dependent process. EETs can also undergo chain-shortening or elongation to epoxyhexadecadienoic acids (EHDs) or epoxydocosatrienoic acids (EDTs), respectively.

Following their formation, fatty acid epoxides, such as EETs, exhibit a range of biological activities and metabolic fates (Fig. 2). These compounds exert their effects by interacting with membrane receptors, or by directly binding to ion channels and other cellular proteins. A significant route for EET metabolism is hydrolysis to dihydroxyeicosatrienoic acids (DHETs) through the action of epoxide hydrolases (EHs) [87]. DHETs often show reduced activity in biological assays [31,88, 89]; however, some physiological processes, including vasodilation and peroxisome proliferator-activated receptor (PPAR) agonism, are similarly affected by EETs and their corresponding DHETs [30,90–92]. Hydrolysis facilitates elimination of EETs since DHETs are released from cells and are not as readily reincorporated into membrane phospholipids [93]. Both EETs and DHETs are found in blood, but only DHETs are detectable in urine, which suggests a process of selective elimination [30,94]. In mammals, two enzymes regulate nearly all EpFA hydrolysis [29]. sEH, also known by its gene name epoxide hydrolase 2 (EPHX2), shows highest activity toward EETs [95]. mEH (EPHX1) is far slower in EET metabolism; however, due to abundant and broad expression and membrane localization adjacent to P450s, mEH also contributes significantly to EET metabolism *in vivo* [29]. Together, mEH and sEH hydrolyze nearly all EpFAs *in vivo* [29]. Genetic disruption or pharmacological inhibition of sEH significantly reduces EpFA hydrolysis and increases EpFAs, most notably the levels of the hallmark sEH substrates 14,15-EET and 12,13-EpOME [29]. Considering the encouraging preclinical evidence supporting the cardiovascular benefits of P450-derived EETs, pharmacological inhibition of sEH presents a promising therapeutic strategy [96–98].

EETs are readily incorporated into the phospholipids of cell membranes across various tissues, including the heart, liver, and kidneys [38, 99,100]. This esterification process is coenzyme A (CoA)-dependent, and EETs are primarily integrated at the sn-2 position of phospholipids [93,101–103]. Interestingly, arachidonic acid (AA) is preferentially incorporated into membranes over EETs. Among the various EET regioisomers, 5,6-EET shows the highest level of membrane incorporation, followed by 8,9- and 11,12-EET, with 14,15-EET exhibiting the lowest incorporation. Membrane esterification suggests that phospholipids may serve as a reservoir for EETs, allowing for their subsequent release and sustained biological activity [104].

Notably, esterification of EETs is likely a robust process; the half-life of PUFAs given by intravenous injection is as little as 3.8–30 s as free PUFAs are rapidly absorbed into tissues and esterified to phospholipids [105,106]. A study that intravenously injected radiolabeled 14,15-EET into dogs observed rapid disappearance of EETs from plasma (t_1/2_–30 s) with only a small fraction (~2 %) ever recovered as plasma or urinary DHETs [107]. Lacking any evidence that esterified EETs are important signaling molecules, these studies suggest that EET esterification is a fast and physiologically relevant process for the storage of EETs and termination of EET signals [103,104,107].

Other metabolic fates of EETs have been observed [108]. EET chain shortening or elongation initiates with acyl-CoA ligation, after which EETs may be elongated to 22-carbon epoxides or shortened through β-oxidation to 16-carbon epoxides that can be reincorporated into plasma membranes and/or maintain physiological activity [102, 109–111]. EETs can be conjugated to glutathione, although it is unclear if this is a physiologically relevant process [112]. EETs binding to fatty acid binding proteins (FABPs) inhibits EH-mediated hydrolysis and/or facilitates EET trafficking [113,114]. EETs can also undergo further metabolism by P450 or COX enzymes; EETs can undergo ω-hydroxylation by CYP4A enzymes [115] and 5,6-EET can serve as a substrate for COXs to yield epoxy prostaglandins [116,117].

### 3.5. Metabolism of other PUFAs

P450-dependent oxidation of AA to EETs and HETEs is the canonical pathway most often studied with regard to PUFA metabolism; however, P450s, can utilize other PUFAs as substrates. P450s also metabolize linoleic acid (LA), and the omega-3 fatty acids epoxyeicosapentaenoic acid (EPA) and docosahexaenoic acid (DHA) to epoxy, dihydroxy and hydroxy oxylipins that have important physiological functions [118, 119] (Table 2). Though less exhaustively studied, it seems likely that the same subset of P450s that have been shown to metabolize AA, also metabolize LA, EPA and DHA. P450s show differential preference for PUFAs. For example, CYP2C8 metabolizes LA three times as fast as AA, while CYP2J2 metabolizes AA twice as fast as LA [53,120]. *In vivo*, plasma levels of LA- and DHA-derived EpFAs are found in concentrations up to several orders of magnitude higher than their corresponding AA-derivatives [29,121].

P450s metabolize LA to either 9,10- or 12,13-epoxyoctadecamonoenoic acids (EpOMEs). Treatments with 9,10- and 12,13-EpOME revealed cytotoxic effects on cells and these effects may be enhanced by hydrolysis to the corresponding 9,10- and 12,13-dihydroxyoctadecamonoenoic acids (DiHOMEs) [122]. Thus, in contrast to the generally beneficial effects of AA epoxygenase products (described below), epoxygenation of LA often has deleterious effects. Many P450s metabolize eicosapentaenoic acid (EPA) and docosahexaenoic acid (DHA) at rates that are similar to or higher than those for AA [123,124]. CYP2C8 and CYP2J2 demonstrate selectivity for epoxygenation of the ω-3 olefin to produce primarily 17,18-eicosatetraenoic acid (EpETE) and 19, 20-eicosadocosapentaenoic acid (EpDPA) [119,124–126]. ω-Hydroxylases also efficiently metabolize EPA and DHA to 19- and 20-hydroxyeicosapentaenoic acids (HEPEs), and 21- and 22-hydroxydocosahexaenoic acids (HDHAs), respectively [119]. Interestingly, several CYPs, including CYP1A1, CYP1E1, CYP4A1, and CYP4A14A that act primarily as hydroxylases toward AA as substrate, have predominantly epoxygenase activity with EPA and DHA as substrates [119,127–129].

## 4. Cellular and physiological targets of EpFA metabolites

Many studies suggest that EETs may signal through a high affinity G protein-coupled receptor (GPCR) [130]. EETs induce physiological responses at low concentrations (1–100 nM), display high-affinity membrane binding of 14,15-EET (Kd 14–35 nM) [131,132], induce GTP-loading of Gαs, and activate cAMP and PKA pathways at low concentrations [40]. Furthermore, experiments with silica-linked EETs demonstrated extracellular binding and activity, implying a cell surface receptor [133]. However, the direct identification of a putative EET receptor (EETR) has proven to be a considerable challenge. Extensive screening efforts using various direct and indirect methods have largely been unsuccessful. Broad GPCR panels have failed to identify a high-affinity EETR, often showing only low-affinity interactions requiring micromolar EET concentrations [134,135]. EETs interact with several GPCRs that may mediate some of their known biological effects, though most of these interactions occur at micromolar concentrations. Identified low-affinity EET receptors include free fatty acid receptors (FFARs) GPR4 (FFAR1), GPR120 (FFAR4), and GPR132 [136–138]. GPR39 responds to sub-micromolar EET levels; however, the unusual effects elicited by EETs through GPR39 suggest it is not the universal EETR [139]. EETs may act as selective antagonists of thromboxane receptors (TP receptors) [140–142] or as agonists through the EP2 and EP4 PGE2 receptors (EP2 and EP4) [143–145]. While several promising leads have been developed, there is no conclusive evidence of a low-affinity GPCR that regulates EET or sEH-mediated physiology.

The cellular targets of other EpFA-derived oxylipins, including EpOMEs, DiHOMEs, EpETEs and EpDPAs are less well studied. Little is known about how EpOMEs and DiHOMEs induce inflammation or cellular proliferation, although the existence of an EpOME or DiHOME-responsive GPCR remains possible. Both EpOMEs and DiHOMEs stimulate neurons to enhance pain sensation through what may be direct binding to transient receptor potential (TRP) channels TRPV1 or TRPA1 [146]. EPA-derived 17,18-epoxyeicosatetraenoic acid (EpETE) was recently identified as a high affinity ligand that can transactivate the sphingosine-1-phosphate receptor-1 (S1PR1) at 10–100 nM concentrations [147]. This activation appears distinct from EET signaling as the putative EET receptor antagonist 14,15-Epoxyeicosa-5(Z)-enoic acid (EEZE) fails to block 17,18-EpETE-induced vasodilation [148]. Like EETs, 19,20-EpDPA also binds to FFARs, though this is not a high-affinity interaction [149].

### 4.1. Hypertension

Several endothelial-derived factors, including nitric oxide, prostanoids and EETs can signal to vascular smooth muscle cells (VSMCs) to induce vasodilation [150]. Though little is known about the expression of endogenous P450 isoforms in hypertension, pharmacological sEH inhibition, genetic sEH disruption or transgenic endothelial overexpression of CYP2C8 or CYP2J2 can attenuate hypertension in mouse models [151,152]. Shear stress can lead to formation and/or release of EETs that act as endothelial-derived hyperpolarization factors (EDHFs). Less than 100 nM of several EETs can result in vasodilation of isolated vessels through the opening of large-conductance Ca^2+^-activated potassium channels (BKCa) in VSMCs [30,153]. BKCa channel opening increases influx of potassium and hyperpolarizes the plasma membrane that ultimately limits calcium influx required for smooth muscle contraction [154]. Since EETs do not directly activate BK_Ca_ channels, two competing hypotheses have been proposed: (a) EETs may activate the putative EETR and Gαs/PKA signaling to increase BKCa opening; or (b) EETs may increase the opening of TRPV4 channels that induce small calcium transients (sparklets) that activate BKCa channels to hyperpolarize cells and diminish intracellular calcium levels [155]. While an initial study showed that 14,15-EET induced vasodilation was fully intact in aortic rings from TRPV4 knockout mice [140], several subsequent studies suggest that TRPV4 and/or TRPC1 channels are critical for EET-induced vasorelaxation [156–158].

P450-derived EETs may also attenuate hypertension through regulation of renal vascular tone, salt handling, and inflammation. EDHF effects of EETs dilate renal arteries and afferent arterioles to increase glomerular flow [92]. EETs activate ERK [159] or PKA [160] which can phosphorylate and inhibit the epithelial Na + channel (ENaC) in distal tubules to increase urinary salt excretion [161]. Both effects could result in decreased blood volume and serve as a second mechanism through which EETs attenuate hypertension. Disruption of *Cyp2j5*, which is abundantly expressed in renal tubules, induces mild hypertension in mice [162]. Though less well-studied, 17,18-EpETE and 19,20-EpDPA act as EDHFs to potently induce vasodilation in blood vessels in a manner similar to EETs [148,163]. In contrast, endothelial expression of CYP2C8 results in increased formation of DiHOMEs and reactive oxygen species (ROS) that lead to vasoconstriction of coronary arteries during reperfusion after ischemia [164].

### 4.2. Angiogenesis, cancer and tissue healing

Low oxygen conditions (hypoxia), such as those that exist in growing tumors, increase expression and activity of CYP2C enzymes in endothelial cells, leading to an increased generation of EETs [165]. EETs are well-known for their significant roles in promoting cell proliferation, migration, and the formation of new blood vessels (angiogenesis) [166, 167]. EETs can transactivate various growth factor receptors, including epidermal growth factor (EGF), vascular endothelial growth factor (VEGF), and basic fibroblast growth factor (bFGF) which trigger downstream effector pathways like PI3K/AKT, MAPK, Rac, or Src, ultimately driving endothelial cell proliferation, migration, and angiogenesis [166,168–172]. Consequently, induction of P450s such as CYP2Cs are implicated in various angiogenesis-dependent physiological processes, such as wound healing, organ regeneration, and even the progression and spread of tumors [169,170,173]. In addition, human cancers display increased levels of CYP2J2 relative to healthy tissues which correlates with enhanced tumor growth and metastasis [174, 175]. Similar to their effects on endothelial cells, EETs contribute to the proliferation and migration of tumor cells [169,170,175,176]. Furthermore, in both endothelial and cancer cells, EETs demonstrate anti-apoptotic properties, protecting cells from programmed cell death induced by various internal or external cues [177–179].

Emerging evidence supports the role of CYP2C- or CYP2J-induced EpOME formation in tumor promotion [180]. Metabolomics analyses revealed that levels of colorectal *Cyp2c* isoforms and EpOMEs are significantly increased in colorectal tumor bearing mice. Furthermore, EpOME treatment exacerbates, while *Cyp2c*-disruption attenuates the development of colorectal cancer in mice [181]. Linoleic acid-rich diet also increases EpOME levels and colorectal tumor development in a CYP2C-dependent manner [182]. EpOMEs activate NF-kB and expression of chemokines such as CXCL9 to induce tumor inflammation, growth and metastases [183].

### 4.3. Inflammation

Overexpression of CYP2C or CYP2J isoforms induce EET formation and attenuate inflammation in a variety of tissues including in endothelial, myocardial and renal tubule cells [184–186]. EETs play a crucial role in attenuating vascular inflammation and remodeling by inhibiting the activation of endothelial cells and reducing the adhesion of leukocytes to the vascular wall [31,187]. These effects appear to be largely due to inhibition of NF-kB activation, subsequent to EET activation of PPARγ [184,188] in processes reviewed elsewhere [189]. Expression of P450 isoforms is typically suppressed early in inflammation [190,191], which supports a paradigm that suppression of P450 epoxygenase activity and anti-inflammatory EETs allows for efficient activation of inflammatory processes. P450 expression is later restored to reintroduce anti-inflammatory EETs that initiate resolution of inflammation [187]. Indeed, the inability to suppress EETs early during infection suppresses macrophage activation and delays pneumococcal clearance from lungs of mice [192].

In contrast, EpOMEs and/or their corresponding DiHOMEs induce cytotoxicity, inflammation, and cytokine release [122,183,193], although it remains unclear what proximal signaling steps induce these effects. It is somewhat paradoxical that the same P450s produce anti-inflammatory EETs and pro-inflammatory EpOMEs. It is possible that CYP2C isoforms are more likely pro-inflammatory than CYP2J isoforms; CYP2Cs show a higher preference for LA over AA as a substrate and are known to produce ROS during their catalytic cycle [53,194].

### 4.4. Ischemic protection

The protective effects of EETs against ischemic injury are well-documented in both cardiac and cerebral contexts. In humans, CYP2J2, which is highly expressed in cardiomyocytes, is thought to be the primary producer of EETs [38]. Increasing cardiac EET levels through transgenic CYP2J2 overexpression, genetic disruption of sEH, or direct EET administration significantly improves functional recovery and limits tissue necrosis following cardiac ischemia [52,97,195,196]. This cardioprotection largely stems from the ability of EETs to preserve mitochondrial integrity in cardiomyocytes during ischemia-reperfusion. EETs activate signaling pathways such as PKA, MAPK, PI3K/AKT, and PKC that lead to the inhibitory phosphorylation of glycogen synthase kinase 3 beta (GSK3β) which curtails the catastrophic opening of the mitochondrial permeability transition pore (mPTP) [52,97,197,198] and preserves the electron transport chain [197,199]. EETs also activate sarcolemmal (sarcKATP) or mitochondrial (mitoKATP) KATP channels as part of their cardioprotective effects. SarcK_ATP_ activation shortens cardiac action potential duration and reduces calcium overload during ischemia [200], while mitoKATP channels protect mitochondria against ischemic damage by unknown mechanisms [97,201]. The precise mechanisms by which EETs engage these channels, potentially involving partial mitochondrial depolarization, transient swelling, reduced calcium overload, or altered ROS production, remain to be fully elucidated [201].

LA-derived EpOMEs and/or DiHOMEs appear detrimental to cardiac function. Intravenous infusion of EpOMEs induces cardiac failure [202], possibly due to coronary vasoconstriction and/or depression of cardiomyocyte respiration and contractility [203,204]. DiHOME infusion suppresses coronary vasodilation and recovery of contractile function of isolated hearts during reperfusion after ischemia [164]. Given the higher propensity of CYP2Cs to metabolize LA than AA [53], these findings might help explain the paradoxical finding that CYP2C-selective inhibitors improve cardiac recovery after ischemia [205,206].

EETs similarly attenuate damage from cerebral ischemia. Elevating EET levels enhances cerebral blood flow during cerebral infarction through both neurogenic and endothelial-mediated vasodilation [207–209]. In the context of stroke, EETs exhibit a broad spectrum of beneficial effects, including vasodilation, neuroprotection, enhanced angiogenesis, and suppression of oxidative stress and post-ischemic inflammation [168,210–212]. Moreover, in addition to their regulatory role in ischemic injury in animal models, EETs contribute to resolving thrombotic blockages in arteries *in vivo*. EETs effectively prevent platelet aggregation independently of thromboxane A_2_ (TXA_2_) synthesis [168, 210–213]. EETs induce membrane hyperpolarization and reduce Ca^2+^ entry into platelets, thereby inhibiting platelet activation, cytoskeletal rearrangement, aggregation, and adhesion to endothelial cells [214, 215].

## 5. Conclusions

The cytochrome P450 enzyme system is a critical contributor to PUFA metabolism, generating a diverse array of bioactive eicosanoids, including AA-derived EETs and HETEs. Specific P450 isoforms, such as human CYP2C8, CYP2C9, and CYP2J2, exhibit distinct epoxygenase activities, yielding EETs with potent effects on vascular function, inflammation, and cellular proliferation. Beyond AA, P450s also metabolize other PUFA including LA, EPA and DHA to producing a complex lipidome with diverse, and sometimes opposing, biological effects. A deeper understanding of P450-mediated PUFA metabolism, including the intricate regulation of enzyme expression and activity, and the precise signaling mechanisms of their products, is essential for identifying novel therapeutic targets and strategies for a wide range of human diseases.

## Figures and Tables

**Table 1:** Cytochromes P450 known to metabolize arachidonic acid (AA) to EETs. P450s, species, Vmax (in pmol/nmol P450/min), percentage of epoxides produced, major expression profile (localization) and references are shown. In some instances, EET formation was observed but not quantitatively measured (Yes). In other instances, qualitative assessment of rates (Low or High) was inferred from manuscripts. ND, not determined.

P450	Species	AA Vmax	% Epoxides	Localization	Reference
		(pmol/nmol/min)			
CYP1A2	Human	60	41 %	Liver, Lung	Bylund 1998
CYP2B (6)	Human	17	2	Liver, Kidney, Intestines	Rifkind 1995
CYP2C8	Human	300–700	50 %	Liver, Extrahepatic	Rifkind 1995, Daikh 1994
CYP2C9	Human	600–1300	62 %	Liver, Extrahepatic	Rifkind 1995, Bylund 1998, Daikh 1994, Imaoka 2005
CYP2C19	Human	200–2000	54 %	Liver, Intestines	Bylund 1998, Daikh 1994, Imaoka 2005
CYP2E1	Human	50	<10 %	Liver, Extrahepatic	Rifkind 1995, Bylund 1998
CYP2J2	Human	65–450	75 %	Liver, Extrahepatic	Wu 1996, Imaoka 2005, Graves 2013
CYP2S1	Human	Yes	ND	Intestines, Extrahepatic	Frömel T, 2013
CYP2W1	Human	Low	~80 %	Fetal, Neoplastic	Zhao 2016
CYP3A4	Human	400	37 %	Liver, Intestines	Bylund 1998
CYP4X1	Human	90	75 %	Broad Expression	Edin, unpublished
CYP2B19	Mouse	Yes	55 %	Skin	Keeney 1998b
mCYP2Cs	Mouse	150–5150	>50 %	Liver, Intestines	Luo 1998, Tsao 2000, Delozier 2004
mCYP2Js	Mouse	22–167	33–81 %	Liver, Extrahepatic	Graves 2013
CYP1A1/2	Rat	High	10–20 %	Liver, Extrahepatic	El-Sherbeni 2014
CYP2B1	Rat	Low-1500	>90 %	Lung	Imaoka 2005
CYP2B12	Rat	Yes	80 %	Skin	Keeney 1998a
CYP2C6	Rat	High	30 %	Liver, Extrahepatic	El-Sherbeni 2014
CYP2C11	Rat	High	60 %	Liver, Extrahepatic	El-Sherbeni 2014
CYP2C23	Rat	~4000	~90 %	Kidney, Liver, Extrahepatic	Holla 1999, Imaoka 2005
CYP2J3	Rat	Yes	ND	Liver, Heart, Extrahepatic	Wu 1997, Imaoka 2005
CYP2J4	Rat	Yes	ND	Liver, Heart, Extrahepatic	Imaoka 2005
CYP2B4/5	Rabbit	Yes	ND	Lung, Kidney Liver	Zeldin 1995
CYP2C1	Rabbit	Yes	64 %	Liver	Laethem 1992
CYP2C2	Rabbit	Yes	100 %	Liver	Laethem 1992

**Table 2:** Oxylipin products listed by enzymatic pathway and substrate PUFA. Names of oxylipins are listed in a grid based on enzymatic pathway (COX, LOX or P450) and PUFA substrate (AA, LA, EPA or DHA). Both COX and LOX pathways can produce 18-HEPE and 17-HDHA, which can serve as precursors to specialized pro-resolving mediators. All 3 pathways can produce many additional midchain hydroxylated oxylipins (not shown).

	COX	P450	P450/EH	LOX
AA	PGD_2_	14,15-EET	14,15-DHET	15-HETE
	PGE_2_	11,12-EET	11,12-DHET	12-HETE
	PGF_2α_	8,9-EET	8,9-DHET	5-HETE
	PGI_2_	5,6-EET	5,6-DHET	LTB_4_
	TXA_2_	20-HETE		Lipoxin A_4_
	11-HETE	19-HETE		Lipoxin B_4_
	15-HETE	18-HETE		
		17-HETE		
		16-HETE		
		15-HETE		
		12-HETE		
		11-HETE		
		8-HETE		
		5-HETE		
				
LA		12,13-EpOME	12,13-DiHOME	13-HODE
		9,10-EpOME	9,10-DiHOME	9-HODE
		13-HODE		
		9-HODE		
				
EPA	PGD_3_	17,18-EpETE	17,18-DiHETE	18-HEPE
	PGE_3_	14,15-EpETE	14,15-DiHETE	
	PGF_3α_	11,12-EpETE	11,12-DiHETE	
	PGI_3_	8,9-EpETE	8,9-DiHETE	
	TXA_3_	5,6-EpETE	5,6-DiHETE	
	18-HEPE	20-HEPE		
				
DHA	17-HDHA	19,20-EpDPA	19,20-DiHDPA	17-HDHA
		16,17-EpDPA	16,17-DiHDPA	
		13,14-EpDPA	13,14-DiHDPA	
		10,11-EpDPA	10,11-DiHDPA	
		7,8-EpDPA	7,8-DiHDPA	
		22-HDHA		

## Abbreviations:

EEZE: 14,15-Epoxyeicosa-5(Z)-enoic acid
K_ATP_: ATP-sensitive potassium channels
bFGF: basic fibroblast growth factor
COX: cycolooxygenase
CPR: cytochrome P450 reductase
DHET: dihydroxyeicosatrienoic acid
DIHOME: dihydroxyoctadecamonoenoic acid
DHA: docosahexaenoic acid
EETR: EET receptor
EpDPA: eicosadocosapentaenoic acid
EPA: eicosapentaenoic acid
EpETE: eicosatetraenoic acid
EGF: epidermal growth factor
EET: epoxyeicosatrienoic acid
EpOME: epoxyoctadecamonoenoic acid
FABP: fatty acid binding protein
FFAR: free fatty acid receptor
GPCR: G protein-coupled receptor
GSK3β: glycogen synthase kinase 3 beta
HDHA: hydroxydocosahexaenoic acid
HEPE: hydroxyeicosapentaenoic acid
HETE: hydroxyeicosatrienoic acid
BK_Ca_: large-conductance Ca2+-activated potassium channels
LA: linoleic acid
mEH: microsomal epoxide hydrolase
mitoK_ATP_: mitochondrial
mPTP: mitochondrial permeability transition pore
POR: P450 oxidoreductase
PPAR: peroxisome proliferator-activated receptor
PLA_2_: phospholipase A_2_
AA: arachidonic acid
PUFA: polyunsaturated fatty acid
ROS: reactive oxygens species
sarcK_ATP_: sarcolemmal
sEH: soluble epoxide hydrolase
TRP: transient receptor potential
VEGF: vascular endothelial growth factor
VSMCs: vascular smooth muscle cells
